# “I” calling in group: How collaborative crafting and LMX augment team performance

**DOI:** 10.1371/journal.pone.0355477

**Published:** 2026-08-12

**Authors:** Shazia Humayun, Sharjeel Saleem, Muhammad Ali Hussain, Rizwan Shabbir

**Affiliations:** 1 Department of Business Administration, Baba Guru Nanak University, Nankana Sahib, Pakistan; 2 Lyallpur Business School, Government College University Faisalabad, Faisalabad, Pakistan; 3 Business School, NingboTech University, Ningbo, China; Western Sydney University, AUSTRALIA

## Abstract

Drawing on Lewin’s Field Theory, this article attempts to explicate the impact of living a calling on team performance via the mediation of collaborative crafting. In doing so, we examine the intricacies of leader-member exchange (LMX) as a boundary condition in these relationships. LMX has nuances in research, with follower outcomes considering individual attributes as predictors. Data collected through questionnaires (N = 198, teams = 49) from Pakistani manufacturing workers were used for analysis. We found that employees’ calling in work units augmented the whole team’s performance with the intervening role of the self-managing strategy of job crafting; moreover, LMX strengthened this relationship. Shifting from the individual paradigm to the team paradigm, we study the role of calling in shaping team performance. This study makes three significant and novel contributions: it extends calling research to the team level, identifies collaborative crafting as the mechanism linking individual calling to team performance, and establishes LMX as a boundary condition that strengthens this indirect effect. Together, these contributions move calling research beyond its previously individual-centric focus toward a team-level, boundary-conditioned account of how meaningful work translates into collective performance.

## Introduction

Presently, organizations are persistently confronted with challenges in a dynamic and competitive milieu, which engenders complexity and uncertainty within the workplace and fosters diversity in the labor force. Individuals’ dispositional attributes are the major stimuli that give a person a more profound sense of meaning, purpose, and intrinsic motivation to perform in a particular direction. “Why work” has many reasons that drive an individual to a better life. These range from “working to live” to “living to work” to satisfy dissimilar needs. Many people feel they have been called by their work and enjoy their tasks and responsibilities [1]. They even find themselves happier serving others, which leads to their subjective gratification. Living a calling is a noteworthy driving force that leads to significant individual and organizational outcomes [2].

The notion of calling has been investigated in literature in different contexts and with different conceptualizations. It is prosocial in nature, has personal values and meaning [3], and can be discussed independently of specific needs [4]. Living a calling has a debated connotation in literature; we here operationalized the concept of calling as ‘meaningfulness in work,’ consistent with the approach of Dik and Duffy [4]. Individuals cannot work in isolation; they need people to interact with, a leader to direct them, and coworkers to share ideas and the ultimate feelings of accomplishment.

Calling shapes the employees’ behavior psychologically, ultimately becoming the performance source. The concept of calling has been studied in Kurt Lewin’s Field Theory [5]. Drawing on Kurt Lewin’s Field Theory, we investigated the impact of living a calling on team performance mediated through the bottom-up job redesign approach (job crafting). As per this theory, a person’s behavior comprises numerous diverse interactions. [5] proposed that humans are driven by dynamic forces, thoughts, and emotions that affect how they behave in their present state. These interactions are said to follow field principles and are impacted by psychological factors, including how the person views a situation. Such a psychological domain is sometimes referred to as ‘life space’ since it includes the individual and their psychological or behavioral surroundings, as well as any current events that might have an impact on the person’s behaviors or emotions. The dichotomy of an individual’s behavior in a group is the main driver behind drawing scholars’ attention towards calling behavior. In this study, we also propose the moderating effect of leader-member exchange (LMX), responding to the research call raised by Guan and Frenkel [6]. To answer the implied gap in literature, we propose our theoretical framework, which aims to synthesize the mediated moderation model using a more systematic approach.

Job crafting is a mechanism that intrinsically motivates workers to reshape their jobs according to the situation, i.e., personal control, task orientation, and making a social connection with others [7]. Job crafting leads to individual performance; however, we here propose that collaborative crafting leads to team and organizational performance by sharing a common objective [8].

We contribute to the literature on calling in several ways. The literature on calling is dominated by individual-level research; however, team-level phenomena still need to be explored, as the research paradigm has shifted from the individual to the team level. In this vein, we aim to identify the patterns of causality at the team level in a different context [9]. We propose that groups or teams are encouraged by both between and within opportunities that shape positive behaviors and lead to team performance. The contextual factors surrounding individual interactions strongly affect individual calling behavior.

Although prior research has demonstrated that living a calling has positive effects on individual outcomes such as work engagement, job satisfaction, well-being, and career commitment, there is a limited body of evidence clarifying how individual calling relates to team-level outcomes. This study is a response to the literature call, as recent studies highlight the dearth of literature on this stream. Recent literature notes that “current literature lacks research that elucidates the specific impact of leaders on employees’ autonomous job behaviors and career development within the Human Resource Development” [10, p. 71]. Past research has focused on living a calling at the individual level, collaborative crafting, and LMX separately, rather than in an integrated, multilevel context. Although the role of groups in shaping employees’ calling experiences has been noted by Buis et al. [9], the behavioral pathways through which individual calling influences team performance have not been empirically studied. Moreover, there is limited empirical evidence from manufacturing teams in developing countries like Pakistan, where interdependence, hierarchical relations, and resource scarcity make collaborative work behaviors more relevant. Recent reviews also highlight that leadership, job crafting, and collective performance remain disconnected lines of research, underscoring the need for integrated multilevel models to understand the role of contextual leadership resources in proactively redesigning jobs and team performance [11]. Thus, the present study attempts to fill this theoretical gap by examining collaborative crafting as a mediator between living a calling and team performance, and by exploring LMX as a contextual boundary condition within the framework of Lewin’s Field Theory. There are limited studies that focus on understanding the relational feedback about living a calling. The premise of our study is that an individual’s meaningful work leads to team performance, mediated by collaborative job crafting. We address this gap explicitly in three ways: firstly, seek out the antecedents of team performance at the individual level; secondly, explore the role of contextual factors that exhibit an auxiliary influence on living a calling; and lastly, examine the group/team-based outcomes of calling. The role of LMX strengthens the behavior of living a calling, and individuals might feel that their work calls them to do something meaningful and craft their job tasks more accurately. The LMX theory has been a dominant framework for delimiting the quality of leader-follower interactive relationships. The significance of this framework is supported by a meta-analysis from Martin et al. [12], who found a direct relationship between the quality of the LMX relationship and follower behaviors. LMX has been studied as a robust predictor of many organization-related outcomes; however, its role as a moderator still warrants more extensive research. LMX depicts the degree of the quality of the relationship between the member and immediate leader, which is dependent upon the belief, admiration, and responsible nature of both.

## Theoretical framework and development of hypotheses

### Theoretical lens

Referring to Lewin’s Field Theory, it is possible to understand, predict, and provide the framework for changing the behavior of both individuals and groups by constructing a “life space” that incorporates the psychological forces that influence people’s conduct at a certain point in time. Lewin first proposed field theory to analyze individual behavior, but eventually, this theory was primarily utilized to analyze and modify group behavior [13].

In the current study, this theory provides an appropriate framework for understanding how employees’ sense of calling influences collaborative crafting and, eventually, team performance. Human behavior is a function of the individual and their surroundings. This highlights that a person’s “life space” includes not only their personal traits but also the organizational, societal, and situational elements that shape them [14].

Similarly, in a high-LMX context, environmental support encourages the expression of living a calling through collaborative crafting, allowing team members to redesign work processes collaboratively, share knowledge, and coordinate effectively. In a work setting, the relationships between leaders and members are crucial in determining whether calling-driven intentions translate into collaborative actions. High-quality LMX relationships offer psychological safety, trust, and mutual respect, which decrease restraining forces in the field and enhance driving forces towards collaborative crafting [15].

This study advances the living a calling literature by demonstrating its influence on team-level performance, not only on individual performance. It also shows that living a calling affects team outcomes, extending beyond personal impact. Additionally, the research contributes to collaborative crafting studies by emphasizing that this behavior involves collaboration between employees and their calling. Furthermore, the study enhances the LMX literature by presenting living a calling as a key contextual resource that strengthens leader-member relationships and facilitates collaborative crafting.

Our review of the literature is organized around three interlocking mechanisms central to this study. First, living a calling functions as an individual-level motivational force comprised of a personal sense of meaning and purpose that shapes how employees approach their work [4]. Second, collaborative crafting represents the shared, socially embedded process through which team members jointly redesign tasks and roles to express this motivation collectively [8,16]. Third, LMX operates as a social and contextual resource depicting the quality of the leader-follower exchange that determines whether individual motivation is translated into collaborative action [17].

### Living a calling and team performance

The notion of calling initially originated from theology, which describes how individuals respond to God’s calling. However, owing to its extended conceptualization, it has managed to attract a good deal of scholarly attention from HR professionals and academics. Based on its varied conceptualizations, ‘living a calling’ continues to present a noteworthy challenge for researchers in the domain of human resource management.

Calling is inherently connected to a sense of significance, purposeful goals, and meaningful engagement within and across teams. Despite external financial incentives, the key factor keeping a person in their contemporary position and reducing turnover is the accomplishment of personal goals. By accomplishing their personal goals, individuals are searching for their calling. Moreover, living a calling has been examined as a source of job satisfaction, work meaning, life satisfaction, and career commitment [18,19]. Living a calling has positive correlations with career [20], well-being [21], and work behavior [22]. Subsequently, individuals who perceive a high calling at work consider work an opportunity and perform it in a challenging way that would arouse other members to perform their tasks with excellence. Committed employees are more enthusiastic, prefer to be at work most of the time [23], and are willing to help their colleagues. Living a calling differs conceptually from work engagement, intrinsic motivation, and job involvement. Work engagement is a positive psychological state in the workplace that is experienced when an individual feels vigor, dedication, and absorption in the work. Intrinsic motivation is the willingness to engage in work activities because they are intrinsically satisfying or pleasurable, and a calling is the experience of seeing one’s work as meaningful, purposeful, and connected to a life mission. Hence, living a calling is not just a passing motivational phase, but a more profound vocational inclination.

The ‘presence’ and ‘searching’ dimensions of calling are fundamental indicators of team performance, which create a desire and a learning spirit among members to ensure innovative and distinctive enactment. Calling orientation tends to make personal and societal contributions by seeing work as purposeful and enjoyable [24]. The individuals’ need for belonging and interaction within the group follows the theme presented by Buis et al. [9], who conducted one of the first studies in which individuals possibly explored their calling within teams [9]. Buis et al. [9] describe ‘blossoms’ as people who have a presence of calling and are feeling the right amount of individuality and belonging in their community. Similarly, ‘authenticators’ do not let one need get in the way of meeting another, but they vary in that they are reaping the benefits of their testing times and evaluation of their vocation [9]. Because their identification motives are balanced, blossoms have found a community with whom they identify their strengths and also feel valued for their distinctive contributions [25]. Individuals who blossom on the team might affirm their own callings while also assisting others in discovering or maintaining their callings, which is a sustained benefit.

According to research, effective mentoring is influenced by both a developed relationship notion [26] and high self-esteem [27]. Blossoms are capable of sharing their knowledge of effective procedures in the hope that it would help others, since they have faced difficult questions and found solutions in their individual and group experiences [28].

At the team level, it has been demonstrated that individuals who have callings view their work as a moral obligation, engage in ethical behavior, and put forth extra effort to uphold high ethical standards [23]. Leaders who exhibit conscientiousness and ethical behavior elicit higher team commitment and team performance from their teams [29]. Lewin’s Field Theory aids in explaining how environmental and human factors influence and maintain high-performance behaviors. Living a calling serves as a powerful internal motivator that drives individuals to engage in collaborative job crafting, a process in which team members collectively shape tasks, relationships, and work procedures to enhance person-job fit and overall effectiveness. When the social field (team environment) promotes autonomy, trust, and respect for one another, people’s basic needs for relatedness, competence, and autonomy are satisfied. However, limiting constraints such as rigid work rules, a lack of trust, or misaligned goals can undermine crafting efforts and reduce their positive benefits on productivity.

Within Lewin’s Field Theory, living a calling operates as a driving force in the individual’s life space, propelling behavior toward purposeful engagement; because life space extends into the shared team environment, this force is expected to manifest directly at the team level as improved performance.

Because living a calling is an individual-level construct while team performance is measured at the team level, this relationship necessarily involves a cross-level, bottom-up process of emergence [30,31]. Individual calling does not translate into team performance as a simple aggregation of independent employees’ states; rather, it does so as employees enact calling-driven behaviors, most notably collaborative crafting. Collaborative crafting, once shared and reinforced across team members, evolves into a collective resource qualitatively distinct from any single member’s contribution. Viewed through Lewin’s Field Theory, individual forces alter the shared team field only once they are enacted and reinforced through interaction among members, not through mere summation of individual states. This composition process explains why an individual-level motivational force can plausibly manifest as a team-level performance outcome, even before accounting for the specific mediating pathway through collaborative crafting.

Due to a dearth of empirical and theoretical investigation on how individual employees’ internal drive, like work calling, stimulates team performance, we propose this relationship in the current study.

***H1.*** *Living a calling is positively associated with team performance.*

### Living a calling and collaborative job crafting

A few decades ago, job design was considered to comprise the activities within organizations that were channeled from top management to employees. Employees are usually bound to follow guidelines and job descriptions, with little autonomy to make changes. The job characteristics model delineates the physical characteristics of a job that motivate individuals from different perspectives [32]. The employees’ motivation depends on the psychological state of the individuals, which internally motivates them to perform the tasks [33], the characteristics and descriptions of the job that motivate employees to complete tasks, and the dispositional features of individuals that lead them to accept challenging tasks. Job autonomy plays an important role in enhancing work engagement and reducing job stress [34]. Theoretical and empirical studies show a significant connection between living a calling and job crafting [35, 36].

Living a calling gives one a worthwhile objective and guides one towards constructive and fulfilling job practices. Drawing on Lewin’s Field Theory, we argue that the influence of calling in life space makes people more anxious about achieving their goals and encourages them to take actions that will get them closer to their ideal condition. In the current study, collaborative job crafting becomes a habit that lessens the conflict between the individual’s ideal, purpose-driven state and the actual work environment [37]. Collaborative crafting transforms the field and enhances team performance by enhancing shared resources like mutual understanding, trust, and shared goals. In contrast to job crafting, where employees independently restructure their own work tasks, collaborative crafting is a process in which team members collectively redesign work activities, coordinate the roles, and adapt workflows to meet team goals. Thus, collaborative crafting involves more than just individual proactive acts and represents a group-level behavioral process. Longitudinal and meta-analytic investigations of job crafting show that collaborative crafting, in turn, cultivates shared social and cognitive resources (coherence, coordination, and trust) that support improved team performance [38]. This is in line with Lewin’s theory that behavioral and environmental changes reinforce one another, and the dynamics of the fields are cyclical.

Research on academic institutions demonstrates that providing students with the chance to work together might help them learn more about scientific reasoning in terms of content and practices. It also assumes that students bring sufficient cognitive and monitoring resources to the classroom, despite the well-documented challenges they face with the complexities of scientific reasoning, to gain through this level of participation. However, with more prior knowledge and training in group scientific reasoning, students could show improved learning results [39] that demonstrate the importance of collaborative working. Si [40] proposed a positive effect of collective efficacy on students’ performance. In this vein, Saleem et al. [41] found a positive impact of collective efficacy on team performance and team commitment. Based on the literature, we argue that individuals’ search and presence of calling take the lead to motivate them to perform challenging tasks in an individual capacity as well as in a collective way. Collaborative crafting would result in the diffusion of positive energies and emotions from individuals to all team members. Their collective goals excite them to combine efforts to accomplish the tasks. Consistent with Field Theory, calling acts as a driving force that increases tension between an individual’s current state and their purpose-driven ideal, motivating collaborative crafting as the behavioral means of resolving this tension within the team’s shared field. So, we propose our next hypothesis as follows:

***H2.*** *Living a calling is positively associated with collaborative crafting.*

### Collaborative crafting and team performance

Collaborative job crafting refers to the collective coordination of work tasks and decisions to achieve mutual goals regarding work redesign [42]. Job crafting has the potential to induce positive work outcomes, e.g., work engagement [43], job satisfaction [44], job performance [45], and sustainable employability [46], among others. Healthy workplace achievements are exhibited through improved psychological ownership that comes through autonomous job redesign [47]. Mäkikangas et al. [48] found a significant moderating role of job crafting in the work engagement-team performance relationship, which might be attributed to the individuals’ sense of empowerment, which encourages them to be more dedicated and complete additional chores with greater engagement at work.

According to Lewin’s Field Theory, shifts in the team’s “social field” have an impact on collective behavior, which explains the relationship between collaborative job designing and team success. The structure and dynamics of the workplace are changed by collaborative job crafting, in which team members work together to reshape tasks, relationships, and workflows. This reduces restraining forces like role ambiguity and siloed work patterns and increases driving forces like shared responsibility, trust, and role clarity. By reshaping the team’s social field in this way, collaborative crafting is expected to translate directly into enhanced team performance.

Professional jobs include a high level of interdependence among team members, involving within-person and between-person task crafting. In interdependent tasks, since the result of the first activity serves as the input for the second, it is illogical to separate the effects of the two tasks. Successful outcomes are hard to reach if a job in the chain is not carried out properly. A good deal of research has dealt with individual workplace outcomes; however, to ensure performance, we emphasize collaborative job crafting in achieving shared goals [49,50]. Empirically, the positive association of collaborative crafting with team performance, interdependence, efficacy, control, and work engagement at the team level is confirmed [8]. Collaborative crafting can create positive norms and practices that can be ingrained over time, or “refrozen” in Lewin’s terms, solidifying high-performance patterns inside the team. Prior to Llorente-Alonso and Topa [51] study, the functions of individual and collaborative job crafting were studied separately at the individual and team levels. However, their arguments have reinforced that individual crafting helps collaborative crafting. According to Leana et al. [16], collaborative crafting results in outputs that are more substantial for the organizations. Leana et al. [16] evaluated the determinants and results of individual and collaborative crafting, and they discovered that the latter enhances organizational commitment and job satisfaction while the former does not [16]. Saleem et al. [52] found that dispositional factors, such as proactive personality, led to both individual as well as collaborative job crafting.

The majority of the work undertaken by people is carried out in teams, even though most of the literature on the calling mechanism is centered on individual characteristics, such as individual traits [53]. Workgroups or teams are collections of people who collaborate on activities together and are socially connected, interdependent, and entrenched in the organizational setting [30]. Given the prevalence of groups in work-related situations and the value of these group memberships to individuals, including work groups in our theoretical and empirical work on calling is relevant [54]. For instance, people commonly rely on their immediate work teams to identify the standards of their business or profession and participate in sensemaking concerning how these beliefs correspond to their innate values and purposes [55]. They frequently make social comparisons with other teammates, assessing their own skills and viewpoints in relation to those of others [56]. These comparisons can highlight or validate a person’s unique skills, talents, and/or abilities [23], as well as their enthusiasm for and interest in a certain position or field [57]. Iida et al. [49], in a recent study, proposed and examined the impact of team job crafting on work engagement in a sample of nurses from Japan. Another recent study by Zhou et al. [58] examined the effect of team job crafting of nurses on their professional quality of life. Based on the above arguments, our third hypothesis is:

***H3.*** *Collaborative crafting is positively associated with team performance.*

### Job crafting as a mediator

The phenomenon of individual job redesigning at the individual level according to their discretion and requirements is known as job crafting [59]. Job crafting denotes the techniques that workers employ with active roles in instigating modifications to the social, physical, or cognitive features of their jobs [60]. Redesigning the job gives employees more freedom to take on creative tasks and engage in social interactions that are advantageous both to the firm and the person. Job crafting is a personal skill that motivates individuals to accomplish their work demands more appropriately. The association between proactive personality and performance has been found to be mediated by job crafting in the empirical literature [52,61]. When work is interdependent across numerous levels, the job may need to be restructured as a result of interpersonal activities, a process known as collaborative crafting. Leana et al. [16] were the first to expand the idea of job crafting as a shared activity or at the team level. Building on the definition of collaborative crafting introduced earlier, we position it here as the proactive mechanism through which individual motivation (calling) is converted into a socially shared work-redesign process. Current studies have sought to understand the significance of personality traits in regulating team effectiveness [e.g., 52,62].

Due to organizational reforms, restructuring, and technological advancement, competitive organizations have lifted proactive behaviors from uniqueness to inevitable needs in contemporary organizations. Practitioners have taken the initiative to broaden the vision of employee job relations and acknowledge the individuals’ needs and roles in reshaping the job boundaries. In our study, the intervening variable is collaborative job crafting as a proactive tool that boosts an individual’s decisive ability to tackle the situation vigorously and boosts the morale of group members to deal with the challenges of job demands. Viewed through Field Theory, collaborative crafting is the behavioral mechanism by which the driving force of calling reshapes the team field, explaining why calling’s effect on team performance operates through, rather than around, collaborative crafting. Therefore:

***H4***. *Collaborative crafting mediates the relationship between living a calling and team performance.*

### Leader-member exchange as a moderator

LMX styles in organizations are most versatile and dynamic in nature as Hughes, Lee, Tian, Newman, and Legood’s [63] findings show that this variable is most widely studied in the literature after transactional leadership in terms of a potent antecedent of employee performance and organizational performance. Wijaya [64] contributes to the literature on high-quality LMX impact on voice behavior moderated by different-gender supervisors. The study by Erdogan and Enders [65] confirms the reciprocal relationship between a leader and their member; if the quality of the relationship is high between leader and member, then employees are more motivated to perform in an organization [66]. This quality of relationship enhances employee performance and organizational citizenship behavior (OCB) [67–69]. The leadership style influences motivation, autonomy, and decision-making of the employees [70,71] and also explains the mechanism as a mediator to enhance members’ creativity [72].

Beyond LMX, leadership approaches such as transformational, servant, and ethical leadership also play important roles in shaping performance outcomes. Gu et al. [72] present a comparative analysis of the effects of transformational and servant leadership on organizational performance, mediated by organizational learning, showing how the combination of leadership characteristics and learning enhances performance. Chaman et al. [71] found a positive effect of ethical and transformational leadership on employees’ introjected motivation. Muterera et al. [73] suggest a strong relationship between job satisfaction and organizational performance by following the perception of both leader and follower. Empirically, it has been shown that an employee’s creativity and innovation depend solely on leadership [63]. The findings of Gashi Tresi and Mihelič [74] demonstrate that high-quality LMX strengthens the relationship between job crafting and self-efficacy [74].

According to theory, a sense of calling is viewed as a personal force that influences motivation and course of action, whilst workplace factors like LMX act as external factors that either support or impede the expression of living a calling. According to this paradigm, LMX serves as a crucial moderating condition: leaders who have strong member-leader relationships offer the resources, psychological safety, and support that enhance the conversion of living a calling into collaborative crafting and, eventually, improved team performance. On the other hand, low-quality LMX may reduce the motivating rewards of living a calling, which would reduce the scope and efficacy of collaborative job crafting. Following Lewin’s planned change approach, organizations can refreeze by integrating these collaborative practices into the team’s norms, unfreeze by identifying and lowering obstacles to collaborative crafting, and change by fostering excellent leader-member relationships and crafting opportunities. This will ensure long-lasting performance improvements. In Field Theory terms, high-quality LMX reduces restraining forces (e.g., ambiguity, distrust) and reinforces driving forces (e.g., psychological safety, autonomy support), thereby strengthening the extent to which calling is converted into collaborative crafting. Therefore, we propose the moderation hypothesis as follows:

***H5.*** *The association of living a calling is positive with collaborative crafting, and this relation is stronger when LMX is high as compared to when it is low.*

## Methodology

### Sample and procedure

One of the researchers gathered data from eight manufacturing firms in Pakistan. After obtaining organizational approval, respondents were recruited from comparable operational divisions across the participating firms. Employees were randomly selected from the available team rosters and invited to participate in the study during working hours. After initial interaction with the staff, the researcher handed out questionnaires to the employees to complete while at work. The objectives of the study were communicated to the participants, and it was made clear that participation was optional and that the data would be treated with the utmost confidentiality. All data were fully anonymized before access by the researchers; the authors had no access to information that could identify individual participants during or after data collection. The research protocol was reviewed and approved by the Ethics Review Committee of the Government College University Faisalabad (Ref. No. GCUF/ERC/664 A). The data collection took place in November and December 2025. The participants provided their written informed consent to participate in this study.

At time 1 (T1), the authors sent ‘living a calling’ questionnaires to the team members, and respective team leaders were contacted at T1 as well to obtain data on LMX. Each team had between three to six subordinates. Only teams with at least three participating members were considered eligible for aggregation and subsequent multilevel analyses. Four weeks later, at time 2 (T2), the authors contacted the same subordinates who responded in wave 1 to get their ratings on collaborative crafting and team performance. The questionnaire, in this way, comprised four parts, i.e., living a calling, collaborative crafting, LMX, and team performance. Living a calling, collaborative crafting and team performance were rated by the subordinates. LMX, however, was rated by the leaders, thus constituting a multilevel model. Initially, the questionnaires were distributed to 300 employees who fell under 68 teams. Respondents were advised to return their completed surveys directly to the author on-site, sealed in the supplied envelope, to maintain confidentiality. Responses were received from 270 employees and 60 supervisors, representing response rates of 90.0% and 88.2%, respectively. Responses were included only when employees completed the required survey waves and could be accurately matched to their corresponding team leader and work team. Responses were excluded if they could not be matched to a specific work group or if they belonged to teams with fewer than three participating members, which did not satisfy the minimum requirement for team-level aggregation. After applying these criteria, the final sample consisted of 198 employees nested within 49 teams and their 49 supervisors, representing effective response rates of 66.0% for employees and 72.1% for teams. Males made up the majority of respondents (56.6%), and most were fairly young (49.8% were between 30–40 years old) and well-educated (58.9% had graduated in advanced technology-related subjects).

### Measures

All variables were measured on a 5-point Likert scale ranging from “1 = strongly disagree to 5 = strongly agree,” unless specified otherwise.

**Living a calling.** The six items of living a calling scale were used to measure this variable. A sample item is: “I am currently working in a job that closely aligns with my calling” [19]. ‘Not applicable’ was also included as an option for participants who had no sense of a calling [19].

**Collaborative crafting.** According to Wrzesniewski and Dutton [59] ideology and scale, items introduced by Leana et al. [16] have been adopted in the current study. Here, we used three items to measure collaborative crafting with a five-point Likert scale ranging from “(1) = not at all to (5) = a great deal.” The items include: “In the past 12 weeks (without supervisory/management input) to what extent have employees (1) changed the skills it uses to make the work more interesting; (2) adjusted the tasks it undertakes to make the job more fulfilling; and (3) changed the variety of work tasks it performs to make the work more meaningful?”

**Leader-member exchange**. This variable is measured by Graen and Uhl-Bien [17] 7-item scale. The items are used to take respondent feedback about how the supervisors support and understand their employees. As the ratings for LMX were provided by the leaders, we used the ‘leader’ referent, and the questionnaire included items such as “How well do you recognize your team members’ potential?”

**Team performance.** In the present study, team performance is measured by a five-point Likert scale developed by Mäkikangas et al. [48], having five items. The sample item is “My team completes the task on time.”

## Results

Considering the hierarchical structure of our data, 198 employees nested within 49 managers, with an average team size of M = 4.04, we utilized multilevel structural equation modeling (MSEM) in Mplus 7.0 [75]. This hierarchical approach also enabled us to address potential common method bias, particularly at the ex-ante (design) stage, as data were collected in two waves from corporate employees and their managers.

### Data aggregation

**Fig 1:**
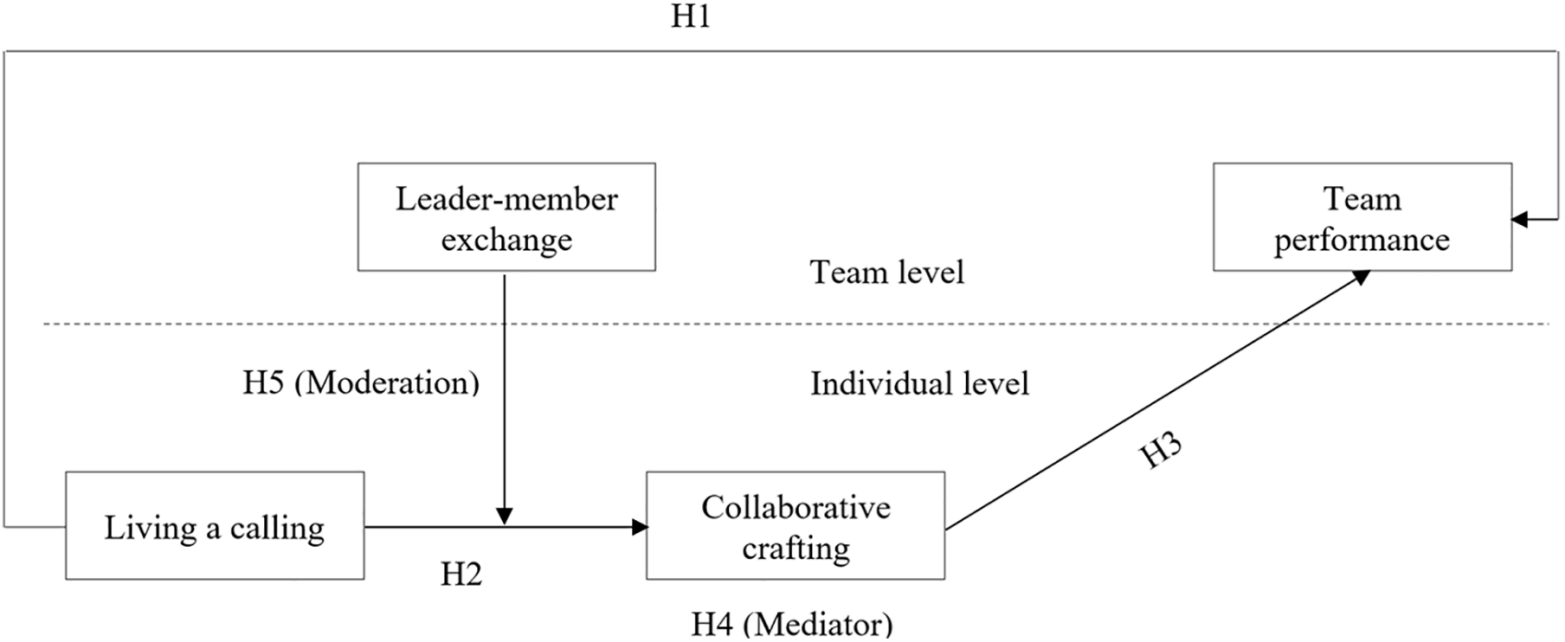
Theoretical framework.

The research model for the present study was constructed at the individual level as well as the team level (see Fig 1). The data for living a calling and collaborative crafting was collected at the individual level, i.e., employees rated their calling and collaborative crafting. The ratings for LMX were obtained from the leaders, thus making it a team-level variable. The ratings for team performance were provided by the individual employees about the performance of their respective teams, which we aggregated to the team level. Therefore, following the guidelines by Kozlowski and Bell [30]; Kozlowski and Klein [31], we first examined whether these individual-rated constructs could be aggregated to the team level. Studies suggest that ICC(1) values greater than 0.05 represent a small to medium effect, while ICC(2) values from 0.40 to 0.75 indicate fair to good agreement, and values above 0.75 signify an excellent agreement [76]. Similarly, the inter-rater agreement values above.70 indicate strong agreement [76]. The aggregation indices for team performance (*ICC(1)* = 0.41, *ICC(2)* = 0.74, r_wg_ = 0.75) indicated that aggregating individual responses at the team level is appropriate.

### Confirmatory factor analysis

The responses to survey items were assessed through multilevel confirmatory factor analysis (MCFA) using maximum likelihood estimation through Mplus 7 [75]. Our results showed that the hypothesized four-factor model fit the data well (χ^2^(174) = 276.768; χ^2^/df = 1.59; CFI = 0.96; TLI = 0.95; RMSEA = 0.055; and SRMR = 0.051). Majority of the factor loadings were significant and higher than 0.70. All the composite reliability (CR) and alpha reliability values were greater than 0.70. The average variance extracted (AVE) values for focal constructs were also above the cutoff value of 0.50. These findings demonstrated the measurement model’s convergent validity. These results are reported in Table 1. To further evaluate discriminant validity and rule out common method bias, we compared the hypothesized four-factor measurement model against a series of increasingly constrained alternative models. A three-factor model and a two-factor model showed poorer fit than the hypothesized model (see Table 2 for full model comparison statistics). These results collectively support the discriminant validity of the four hypothesized constructs.

**Table 1:** Factor Loadings, Cronbach*’*s Alpha (A), Composite Reliability and AVE.

Variables	Loading	Cronbach’s α	Composite reliability	AVE
Living a calling				
Calling 1	0.72^**^	0.91	0.91	0.64
Calling 2	0.84^**^			
Calling 3	0.77^**^			
Calling 4	0.86^**^			
Calling 5	0.81^**^			
Calling 6	0.78^**^			
Collaborative crafting				
Collaborative crafting 1	0.84^**^	0.84	0.84	0.64
Collaborative crafting 2	0.79^**^			
Collaborative crafting 3	0.78^**^			
Team performance				
Team performance 1	0.78^**^	0.91	0.92	0.68
Team performance 2	0.77^**^			
Team performance 3	0.89^**^			
Team performance 4	0.84^**^			
Team performance 5	0.84^**^			
Leader-member exchange				
LMX 1	0.65^**^	0.91	0.92	0.62
LMX 2	0.77^**^			
LMX 3	0.78^**^			
LMX 4	0.63^**^			
LMX 5	0.76^**^			
LMX 6	0.92^**^			
LMX 7	0.95^**^			

*Note(s).* AVE = Average variance extracted; ^**^p < 0.01.

**Table 2:** Comparison of Alternative Measurement Models for Main Constructs.

	Model	Factors	χ^2^(df)	χ^2^/df	RMSEA	SRMR	TLI	CFI	Model Comparison	Δχ^2^	Δdf
1	Hypothesized four-factor model	Living a calling; Collaborative crafting; Team performance; LMX	276.768^**^ (174)	1.59	.055	.051	.95	.96			
2	Three-factor model	Living a calling + Collaborative crafting; Team performance; LMX	440.381^**^ (177)	2.49	.087	0.070	.87	0.89	2 versus 1	163.613^**^	3
3	Two-factor model	Living a calling + Collaborative crafting + Team performance; LMX	610.936^**^ (179)	3.41	0.110	0.084	.79	.82	3 versus 1	334.168^**^	5

** p < 0.01.

Structural relationships were tested using aggregated composite scores for each construct, consistent with common practice in path-analytic mediation-moderation designs; model estimation used the MLR estimator in Mplus 7.0, which provides standard errors and a scaling correction robust to non-normality. The cross-level interaction term (living a calling × LMX) was specified using Mplus’s default latent decomposition of variables in multilevel models; because LMX was measured and modeled exclusively at the team level, the between-level component of this interaction term corresponds to team-mean living a calling interacted with LMX, consistent with recommended approaches for cross-level moderation [77]. Model estimation terminated normally, with no evidence of non-convergence or improper solutions (e.g., negative residual variances).

### Descriptive statistics and correlations

Table 3 shows the means, standard deviations, and correlations among the study’s focal variables. Most of the correlations were significant and in the expected direction, providing preliminary support to our hypotheses.

**Table 3:** Descriptive Statistics and Correlations.

		Mean	SD	1	2	3	4	5	6	7
1	Gender	0.43	0.49	–						
2	Age	1.54	0.51	.002	–					
3	Experience	1.74	0.80	−.080	.319^**^	–				
4	Living a calling	3.12	0.93	.021	−.072	.149^*^	*(.800)*			
5	Collaborative crafting	3.23	0.90	−.117	.033	.199^**^	.444^**^	*(.800)*		
6	Team performance	3.10	1.04	−.021	−.030	.148^*^	.676^**^	.413^**^	*(.825)*	
7	Leader-member exchange	3.46	0.93	.041	−.160^*^	−.208^**^	−.069	−.037	.024	*(.787)*

*Note.* N = 198. Diagonals in parenthesis under Fornell-Larcker criterion represent the square root of average variance extracted (AVE).

* p < 0.05 ** p < 0.01.

### Hypotheses testing

**Fig 2:**
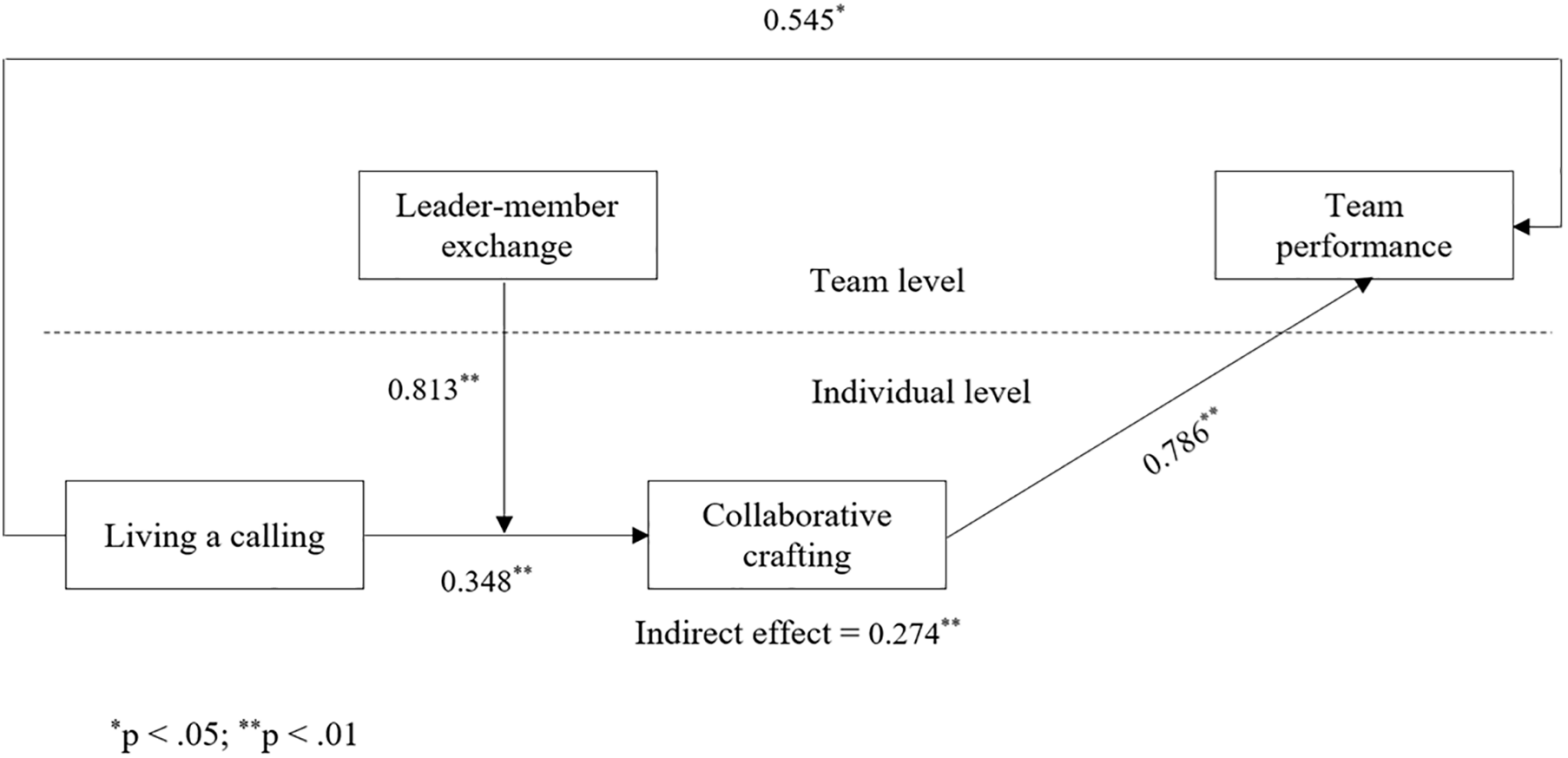
Path estimates.

Multilevel Structural Equation Modeling (MSEM) was employed using Mplus 7.0 to test the hypotheses. The MSEM results are displayed in Table 4 and Fig 2. The findings demonstrated a positive and significant association between living a calling and team performance (β = 0.545, p < .05; H1 supported). Moreover, living a calling positively predicted collaborative crafting (β = 0.348, p < .01; H2 supported). Additionally, there was a positive and significant relationship between collaborative crafting and team performance (β = 0.786, p < .01; H3 supported). Furthermore, the results provided support for H4, revealing a mediation effect of collaborative crafting between living a calling and team performance (indirect effect = 0.274, CI = 0.080; 0.467).

**Table 4:** Structural Model – Hypotheses Test Results.

			Collaborative crafting		Team performance_Level2_	
Living a calling			0.348^**^ (0.096)		0.545^*^ (0.253)	
LMX_Level2_			-0.554^**^ (0.066)			
Living a calling*LMX_Level2_			0.813^**^ (0.047)			
Collaborative crafting					0.786^**^ (0.184)	
***Indirect effect***			Effect size	SE	LLCI	ULCI
Living a calling → Collaborative crafting → Team performance_Level2_			0.274^**^	0.099	0.080	0.467
**Conditional *direct* effect of living a calling on collaborative crafting (moderator: LMX_Level2_)**						
	Effect size	SE	LLCI		ULCI	
LMX_Level2_						
Mean - 1SD	0.208	0.110	0.027		0.389	
Mean	0.359	0.094	0.204		0.515	
Mean + 1SD	0.511	0.091	0.362		0.660	
**Conditional *indirect* effect of living a calling on team performance_Level 2_, via collaborative crafting**						
	Effect size	SE	LLCI		ULCI	
LMX_Level2_						
Mean - 1SD	0.221	0.200	-0.107		0.55	
Mean	0.382	0.249	-0.027		0.791	
Mean + 1SD	0.543	0.304	0.043		1.042	
Index of moderated mediation	0.161	0.065	0.055		0.267	

*Note.* N = 198; SE = standard error; LLCI = lower limit confidence interval; ULCI = upper limit confidence interval.

Standard errors are given in parentheses.

* *p* < .05; ** *p* < .01.

### Moderation analysis

**Fig 3:**
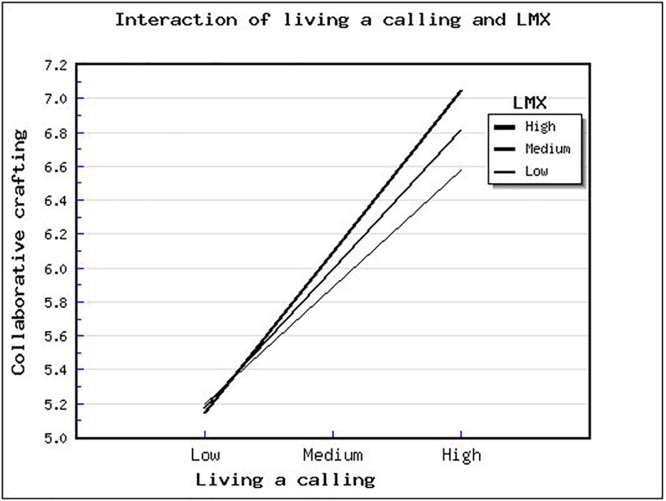
Interaction of living a calling and LMX with collaborative crafting.

Mplus enables the researcher to test the mediation, moderation, and moderated mediation in one structure; therefore, this study relied on Mplus for its model testing [78]. The results in Table 4 reveal that collaborative crafting was significantly affected by the interaction of living a calling and LMX (living a calling*LMX = 0.813, p < .01). These findings indicated that LMX had a significant moderating effect on the link between living a calling and collaborative crafting. Thus, H5 was supported. The moderation analysis results reveal that the positive impact of living a calling on collaborative crafting is enhanced if the employees in a team have higher levels of LMX. It can be seen from the conditional effects and conditional indirect effects reported in Table 4 that as the score of LMX increases, the effect of living a calling on collaborative crafting becomes stronger and more positive. We plotted the interaction effects as shown in Fig 3, which reveals the enhancing of the positive effect of living a calling on collaborative crafting as the score for LMX increases.

Beyond statistical significance, these findings carry substantive meaning for manufacturing teams context. The standardized effect of collaborative crafting on team performance (β = .786) exceeds conventional benchmarks for a large effect [79], indicating that team-level differences in collaborative crafting account for a substantial share of variance in team performance. Practically, this suggests that e ven teams whose members report only moderate levels of living a calling may still achieve marked performance gains if they actively engage in joint task redesign and mutual role adjustment, i.e., crafting behaviors that are arguably more responsive to organizational intervention than an employee’s underlying sense of calling. The interaction effect (β = .813) is similarly substantial in practical terms: the association between living a calling and collaborative crafting nearly doubled in magnitude from low LMX (simple slope = 0.208) to high LMX (simple slope = 0.511). In manufacturing settings characterized by hierarchical supervision and interdependent production tasks, this indicates that the benefits of employees’ calling are unlikely to translate fully into coordinated crafting behavior unless supervisors invest in high-quality leader-member relationships; without such relational support, much of calling’s potential team-level benefit may go unrealized.

## Discussion

This study examined how living a calling shapes team performance through the mediating role of collaborative crafting and the moderating role of LMX, within the framework of Lewin’s Field Theory. Consistent with Field Theory’s premise that behavior emerges from the interaction between the individual and their psychological environment, living a calling, an individual-level driving force, translated into team-level performance only through employees’ enactment of collaborative crafting, the mechanism by which this force reshapes the team’s shared field. This supports Field Theory’s core proposition that individual forces operate through, rather than around, the field. Our findings also extend the theory by showing that this cross-level pathway depends on a specific relational condition (LMX). Thus, we refine Field Theory’s application to multilevel settings. i.e., the “field” is not uniform across a team but is shaped by dyadic leader-member relationships that govern how much of an individual’s driving force converts into collective action.

These findings align with, and extend, prior empirical work across various streams of literature. Prior research has established a positive individual-level association between calling and job crafting Li [35,36]. Our results extend this by showing that the motivational force of calling translates into a collective, team-level crafting process rather than remaining confined to individual redesign. On collaborative crafting and team performance, our findings build on McClelland et al.‘s [8] demonstration that collaborative crafting is a genuinely team-level process distinct from individual crafting. Our findings also align with Llorente-Alonso and Topa [51]’s view that individual and collaborative crafting are interrelated. Situated against the backdrop of the broader team-performance literature, our findings complement rather than compete with other established antecedents: where Mäkikangas et al. [48] identified work engagement and Saleem et al. [41] identified collective efficacy as drivers of team performance. Our results suggest collaborative crafting, motivated by individual calling, operates as a further, distinct pathway to the same outcome, broadening the set of mechanisms through which teams achieve high performance.

On leadership, our moderation findings are consistent with Erdogan and Enders [65] finding that high-quality leader-member relationships strengthen the link between employee motivation and performance-relevant behavior. We extend this logic to a calling-driven, team-level process, showing LMX shapes whether a team-level motivational resource is realized at all, not merely whether individual outcomes improve. This is broadly consistent with Park et al.‘s [29] finding that leader behavior shapes how individual calling translates into team commitment and performance. Taken together, living a calling appears to translate into enhanced team performance only when accompanied by both a behavioral mechanism (collaborative crafting) and a relational context (high-quality LMX).

### Theoretical implications

To our knowledge, this is the earliest empirical study to demonstrate, through the lens of Kurt Lewin’s Field Theory, that collaborative crafting is positively related to team performance and mediates the relationship between individual calling and team performance, moderated by LMX. An Individual’s calling diffuses positive attributes to another group member that would influence the team’s performance.

This study makes three distinct contributions. First, to the calling literature, we shift the unit of analysis from the individual to the team, demonstrating that living a calling is not only a personal resource but also a collective one that shapes team-level performance, extending calling research beyond its predominantly individual-level focus [3,9]. Second, to the collaborative job crafting literature, we identify collaborative crafting as the mechanism that translates an individual’s sense of calling into shared team outcomes, building on evidence that crafting behaviors aggregate into team-level resources supporting collective functioning [8,49]. Third, to LMX and team-performance research, we show that the quality of leader-member relationships conditions whether calling-driven motivation converts into collaborative action, positioning LMX as a boundary condition rather than a direct antecedent of performance. Collectively, grounded in Lewin’s Field Theory, these findings clarify how individual psychological forces (calling) interact with team-level environmental forces (LMX) to shape collaborative behavior and performance.

### Practical implications

Our theoretical typology of individual calling experiences in group settings is intended to spark conceptual exploration, experimental investigation, and useful calling-related suggestions.

Questions of individual need fulfillment connected to living a calling in group situations might become tractable via research on the concepts discussed here. How people, for instance, assess the value of possible groups or concurrent group memberships at work, home, the community, etc., may be evaluated by individual motivation. HR officials ought to create a work context that encourages individuals to craft their jobs and strategically align them with the team’s objectives. Likewise, as professional mentors, leaders might model constructive job crafting activities such as expanding their relational boundaries of work by increasing interactions with their subordinates. The importance of leaders cannot be underestimated since they inspire others to accomplish their duties more effectively. The study’s most significant implication is that it shows how to encourage employee engagement and loyalty, which disseminates positivity throughout the organization.

Grounded in the present findings, several concrete interventions are suggested for manufacturing team settings. There is a need for leadership coaching programs to promote high-quality exchange relationships between employees and their supervisors, given that LMX significantly strengthened the translation of calling into collaborative crafting in our findings. Additionally, HR can also provide collaborative job crafting sessions in teams, where team members will redesign work and collaborate on tasks, directly targeting the mechanism shown here to most strongly drive team performance. Structured mentoring programs and autonomy-supportive work practices could also contribute to a sense of living a calling and to collaborative work performance.

### Limitations and future research directions

Although this study has contributed meaningfully, it has some limitations. First, the study used a two-wave research design, but most measures were self-reported questionnaires, which may cause perceptual bias; this concern is partially mitigated by the fact that LMX was rated by a separate source (team leaders) rather than by the same respondents who reported living a calling, collaborative crafting, and team performance. Second, our sample comprised 49 teams, with an average team size (M = 4.04), which limits statistical power at the team level; caution is therefore warranted in generalizing the precise magnitude of the team-level effects reported here, and replication with a larger number of teams would strengthen confidence in these findings. Third, since the study was conducted in Pakistani manufacturing organizations, the findings cannot be broadly generalized to other manufacturing sectors or different cultural contexts. Fourth, with the four-week time lag, it is difficult to make strong causal inferences, and we interpret our findings as evidence of theoretically grounded associations rather than definitive causal effects. We have already accepted that not everyone is conscious of the idea of living a calling or seeking one, but we would argue that everyone seeks purpose and identity to some extent, which is geared to team performance. How do individuals whose concern about living a calling is more pressing differ from those who are not, and how does it affect the group or team performance? We encourage future researchers to explore these questions with alternative models in a different context and setting. The role of leadership is obvious, and the researchers may take into account different styles to understand numerous antecedents of team performance. Future research should explore other models (such as servant leadership, transformational leadership, empowering leadership and ethical leadership) to determine if they have similar moderating effects. Longitudinal and cross-cultural designs and multisource, objective team performance measures can also be used by researchers on larger multilevel samples from various industries to assess the robustness of the proposed model.

## Supporting information

S1 File Dataset. (CSV)
